# Improving access to integrated community‐based HIV, HCV and harm reduction services for people who inject drugs in Putao district, North Myanmar

**DOI:** 10.1002/jia2.26355

**Published:** 2024-09-12

**Authors:** Nini Tun, Cho Lwin Oo, Cho Myat Nwe, Lutgarde Lynen, Tom Decroo, Frank Smithuis, Tinne Gils

**Affiliations:** ^1^ Department of Clinical Sciences Institute of Tropical Medicine Antwerp Belgium; ^2^ Medical Action Myanmar Yangon Myanmar; ^3^ Myanmar Oxford Clinical Research Unit Yangon Myanmar; ^4^ Department of Family Medicine and Population Health University of Antwerp Antwerp Belgium; ^5^ Mahidol Oxford Research Unit Bangkok Thailand; ^6^ Global Health Institute University of Antwerp Wilrijk Belgium

1

People who inject drugs (PWID) are disproportionally affected by HIV acquisition [1]. Myanmar, a large producer of opium, has an estimated 116,000 PWID, among whom an estimated 26.4% are people living with HIV [2, 3]. Needle sharing contributed to one‐third of the national HIV incidence in 2018 [4]. The national harm reduction programme includes prevention and care for HIV, viral hepatitis C (HCV), other sexually transmittable infections and tuberculosis (TB), needle and syringe exchange (NSE), and opiate substitution therapy (OST) for PWID [4]. Yet, nationally, only 24.0% of PWID were tested for HIV, and 47.8% of HIV‐positive PWID were on antiretroviral treatment (ART) [3]. Even in Yangon, PWID experience barriers to access NSE and OST [5].

Putao is a remote sparsely populated district on the slopes of the Himalayas in the far North of Myanmar [6]. Sources of income include agriculture, and gold mines operated by increasing numbers of migrant workers. Opioid cultivation sites exist [6]. Heroin injecting is common in Putao, among miners, and in rural communities [7]. Access to health services for PWID is tremendously challenging. Poor road infrastructure, lack of public transport and extreme remoteness of the villages hamper physical access. OST is restricted to government hospitals. Like elsewhere, PWID are insufficiently aware about the risks of heroin use, associated blood‐borne infections, and available care [5] and often stigmatized by community members [8].

Before 2012, no PWID‐specific harm reduction services existed in Putao. HIV testing and ART initiation were provided at a public hospital, where only seven ART patients were registered as PWID before 2012.

Medical Action Myanmar (MAM), a medical organization, is present in Putao since 2012. In a first phase, MAM provided clinic‐based primary care services, following a request by a local organization and because no other non‐governmental organizations were present. Due to a lack of key population data and PWID‐specific services, and a suspicion of PWID presenting with advanced HIV, MAM started clinic‐based HIV testing and treatment, while referring TB patients to a local organization for treatment and care. The high incidence of malaria and TB and difficulties with linkage to care prompted MAM to set up a network of community health workers (CHWs) providing malaria, TB and primary healthcare services in remote communities in 2014. CHWs were selected by MAM and village leaders among community volunteers, trained by MAM, and incentivized per diagnosis, referral, and treated malaria or TB patient. Trained CHWs received a joint certificate from the Ministry of Health and MAM. Due to the remoteness of the Putao district, clinic‐based HIV services were insufficient to reach most PWID. Between 2012 and 2017, only 144 PWID were initiated on ART (Table 1).

**Table 1:** Harm reduction services and outcomes after implementation in Putao district, Myanmar between 2012 and 2023

Project phases^a^	First phase		Second phase											
Year	2012−2017		2018		2019		2020		2021		2022		2023	
**Number of integrated community workers**	NA		*n*	%	*n*	%	*n*	%	*n*	%	*n*	%	*n*	%
*N* of CHW (% of all villages with PWID)			13	8	19	12	23	14	23	14	25	15	46	28
*N* of PV (% of all villages with PWID)			4	2	17	10	32	19	41	25	47	29	61	37
**Number of PWID reached with services**	NA		*n*		*n*		*n*		*n*		*n*		*n*	
*N* of PWID accessed harm reduction			1057		1602		2168		2524		2358		2575	
**Injection‐related services**	NA		*n*		*n*		*n*		*n*		*n*		*n*	
*N* of needles and syringes distributed			106,430		570,161		806,983		1,134,150		1,217,000		959,504	
*N* of needles and syringes distributed ppy			101		356		372		449		516		373	
*N* of naloxone injections given			86		88		84		87		84		92	
**HIV‐related services**	*n*	%	*n*	%	*n*	%	*n*	%	*n*	%	*n*	%	*n*	%
*N* tested for HIV (% of all PWID)	^/^		875	83	941	59	1219	56	1380	55	1553	66	1410	55
HIV positive	^/^		374	43	250	27	259	21	200	14	156	10	180	13
*N* initiated on ART^b^	144	^/^	281	75	181	72	237	92	167	84	136	87	160	89
Total on ART at year end^c^	123	85	268		351		547		601		606		664	
Viral loads done	2	1,6	5	1,9	150	43	236	43	350	58	337	56	534	80
Viral loads suppressed	2	100	5	100	141	94	230	97	330	94	319	95	522	98
**HCV‐related services** ^d^	NA	%	*n*	%	*n*	%	*n*	%	*n*	%	*n*	%	*n*	%
*N* HCV Ab tested			705	67	687	43	517	24	564	22	497	21	513	20
*N* HCV Ab positive			477	68	447	65	406	79	428	76	345	69	384	75
*N* HCV RNA tested			0	0	2	0,5	276	68	382	89	54	16	251	65
*N* HCV RNA positive			0	0	2	100	242	88	314	82	39	72	202	80
*N* DAA initiated			0	na	0	na	48	na	316	na	63	na	145	na
*N* sustained virologic response			0	na	0	na	34	71	226	72	32	51	89	61

*Note*: Harm reduction services include injection‐related services, HIV and HCV‐related services and numbers of PWID reached with each service are not mutually exclusive.

Abbreviations: Ab, antibody; ART, antiretroviral treatment; CHWs, community health workers; DAAs, direct‐acting antivirals; HCV, hepatic C virus; na, not applicable; ppy, per person‐year; PVs, peer volunteers; PWID, people who inject drugs; RNA, ribonucleic acid.

^/^ Data unknown as data collection still incomplete.

^a^ Before 2012: No PWID‐specific services were available. HIV testing and ART was only available at the hospital. Between 2012 and 2017: Services were not yet comprehensive. Between 2018 and 2020: CHWs provided NSE, naloxone administration, health education (including for HIV) and referral for HIV counselling and testing and OST. HIV testing could be done by a mobile team, and ART was clinic‐based. From 2020: CHWs started monitoring ART and DAA adherence in PWID, but treatment initiation (ART/DAA) remained clinic‐based. From 2023: CHWs started HIV testing.

^b^ Yearly numbers initiated, not cumulative.

^c^ Cumulative numbers by year.

^d^ DAA initiation may exceed RNA‐positive cases as they may have been positive in the previous year.

In a second phase, from 2018 to 2023, clinic‐based HCV antibody testing was introduced, and HIV viral load was scaled‐up. An adjacent drop‐in centre was added providing psychosocial counselling, a warm meal and a relaxation room with NSE. For methadone‐based OST, PWID were referred to a government hospital. There, they had to present daily for the first 4–12 weeks and could receive 2‐week take‐home doses after a doctor's decision. For legal support, PWID were referred to a local NGO. Gradually, CHWs were trained and engaged to provide NSE, naloxone administration, health education (including on HIV and HCV) and referral for HIV counselling and testing and OST for PWID. They were supported by peer volunteers; PWID who adhere well to harm reduction services, acting as facilitators between CHWs and PWID and providing peer support, health education and NSE. CHWs and peer volunteers received small incentives. A MAM‐staffed mobile medical team with a doctor and PWID peer educators provided mobile medical services, including HIV and HCV testing, and supported and trained the CHWs and peer volunteers. In 2018 and 2019, respectively, 67.7% and 69.4% of tested PWID were positive for HCV antibodies. In 2020, sofosbuvir and daclatasvir/velpatasvir‐based HCV treatment was introduced at the clinic and CHWs and peer volunteers started providing home‐based monitoring of ART and HCV treatment, after clinic‐based initiation. Since 2023, CHWs and peer volunteers also provide HIV testing and counselling. Between 2018 and 2023, 1378 PWID started methadone.

Aside from 2023, NSE distribution per person‐year increased since 2018. The total number of PWID tested for HIV also increased, while proportional testing uptake and HIV positivity peaked in 2018 and declined as more PWID knew their status. ART uptake increased since 2017, staying above 80% since 2020. Viral load coverage gradually increased with viral load scale‐up until 80% in 2023, and viral suppression (≤1000 copies/ml) has remained consistently high. Among 136 PWID who initiated ART in 2022, 119 (87.5%) were still on ART in December 2022. In June 2023, 89 (74.8%) of 119 were still on ART, among them 71 (79.8%) had a viral load result, and 64 (90.1%) of 71 had a suppressed viral load. HIV testing and ART uptake compares favourably with pooled global figures; 48.8% of PWID were tested for HIV and 47.5% of HIV‐positive PWID were on ART in 2022 [3]. HCV testing declined from a 2018 peak, but HCV antibody and HCV RNA positivity remained relatively stable. In 2022, supply problems with HCV RNA tests caused a drop in testing and HCV treatment initiations, partially recovering in 2023. Cumulatively, direct‐acting antivirals (DAAs) were initiated in 572 (71.6%) out of 799 people with detectable HCV RNA. Between 2022 and 2023, less than 70% of PWID on DAA returned for outcome assessment. We did not systematically collect data on HCV re‐infection.

We hypothesize that CHW and peer volunteer proximity to educate and refer PWID contributed importantly to the expansion of HIV and HCV services. Reaching, initiating and retaining PWID on ART is challenging, however. Some PWID moved for financial reasons, or were without stable housing, others lacked motivation to adhere to ART and/or suffered from social discrimination and depression [8]. In the next phase, MAM aims to improve retention by providing more differentiated ART delivery options and welcome‐back campaigns for those re‐entering care. Poor access to OST due to stringent government regulations may have contributed to instability among PWID and poor compliance. MAM advocates for community integrating OST with HIV, HCV, TB and NSE services, which is likely to improve HIV‐related outcomes [9].

While MAM mobile teams cover the whole district, including the gold mines, with harm reduction activities, they are not permanently stationed in the communities. Currently, 46 CHWs and 61 peer volunteers provide community harm reduction alongside their routine activities. In 2024, MAM is training an additional 119 CHWs and 120 peer volunteers to conduct harm reduction activities, covering all 165 communities with PWID, including those in gold mines.

To work towards sustainability, programmatic costs were minimized by adding health services for PWID to the already funded CHW network. Community participation is essential for increased acceptance and stigma reduction. MAM made mobile health services available to the whole community, and undertook community engagement sessions with community members, including those directly or indirectly affected by substance abuse. Eventually, we aim to engage the whole community in the district to support harm reduction services.

We showed that a community‐based model with integrated HIV and HCV treatment and harm reduction services can obtain good health outcomes for PWID in an extremely challenging setting. Long‐term ART outcomes and retention in harm reduction services should be evaluated.

## COMPETING INTERESTS

All authors declare no conflicts of interest.

## AUTHORS’ CONTRIBUTIONS

NT and FS designed and supervised the programme. CLO and CMN performed the programme activities. NT, TG and FS analysed the data. NT and TG wrote the first draft. LL, TD and FS critically reviewed the paper. All authors read and approved the manuscript.

## FUNDING

This work was supported by the United States Agency for International Development (USAID/MAM/CPI‐003) and the Global Fund (20864‐010‐01).

## Data Availability

The data that support the findings of this study are available on request from the corresponding author. The data are not publicly available due to privacy or ethical restrictions.
